# Enablers and barriers to MENA'S path to universal health coverage: A scoping review of UAE, Morocco and Yemen

**DOI:** 10.1016/j.dialog.2026.100291

**Published:** 2026-03-10

**Authors:** Mai Mohamed Abdou Mahmoud, Lorraine Frisina Doetter

**Affiliations:** a University of Bremen, Germany; b University of Bremen, Research Center on Inequality and Social Policy, Mary-Somerville-Straße 5, 28359 Bremen, Germany

**Keywords:** UHC progress, Barriers, UAE, Morocco, Yemen, MENA

## Abstract

The World Health Organization's (WHO) guidance to the Middle East and North Africa (MENA) region, alongside post-Arab Spring legal reforms, has prompted many countries to prioritize Universal Health Coverage (UHC). Despite these efforts, progress remains uneven, and reforms have not consistently translated into improved population health outcomes. While global monitoring data reveal wide variation in UHC advancement, academic analysis of the factors behind these differences—particularly across countries with varying performance levels—remains limited.

This paper addresses this gap through two objectives: first, to identify general enablers and barriers to UHC and assess the usefulness of these categories; and second, to examine how these factors operate in the MENA context. A scoping review, guided by PRISMA-ScR, was conducted using a six-dimension analytical framework. MENA countries were classified as low, middle, and high UHC performers based on the WHO Service Coverage Index (SCI), with one representative country from each category selected for in-depth review.

Findings highlight six dimensions shaping UHC progress—social infrastructure, economic infrastructure, service delivery, governance, health resources, and financing—revealing both shared and context-specific influences. While financial inputs remain critical, strong governance and political commitment are equally essential for advancing coverage, equity, and quality. The study provides a nuanced understanding of UHC progress in the MENA region and offers actionable guidance for designing context-sensitive and sustainable health reforms.

## Highlights

• Although, MENA region is considered homogenous, there are variations across countries' progress towardUHC.
• Governance serves as the foundational entry point for all other policy dimensions, acting as a critical driver of UHC progress.
• The six-dimensions framework of enablers and barriers should be contextualized.

## 1 Introduction

By signing the UHC2030 Global Compact during the Ministerial Meeting on the Road to Universal Health Coverage (UHC), countries in the Middle East and North Africa (MENA) region reaffirmed their commitment to advancing equitable, resilient, and people-centered health systems [1]. This renewed political momentum has prompted both low-middle income countries (LMICs) and high-income countries (HICs) in the region to undertake legislative and policy reforms aimed at accelerating progress toward UHC [2].

Although a growing body of literature examines UHC dynamics in the region (e.g., [2], [3], [4], [5], [6], [7]), most studies treat challenges in broad terms (e.g., identifying ‘weak governance’ or ‘inadequate financing’ without examining how these factors vary across different governance structures or income levels), offering limited granularity on country-specific contexts. Enablers and barriers to UHC are often discussed implicitly, with limited analytical frameworks to distinguish them across varying levels of UHC performance. Few studies have systematically examined these factors across MENA countries by UHC performance.

This paper seeks to address this gap through two core objectives: (i) to apply and validate a structured categorization of enablers and barriers to UHC; and (ii) to examine how these factors manifest within the MENA region through a scoping review of three representative countries—each selected from a high, middle, and low UHC-performance tier. This examination focuses on both socio-economic context in these countries along with systematic factors including stewardship, service delivery and health financing struggles.

The central research question guiding this study is: What enablers and barriers influence the progress of MENA countries across varying income levels toward achieving UHC? To answer this, the paper first classifies countries by Service Coverage Index (SCI) and UHC Index levels, selects one representative country per performance tier, and conducts a scoping review to identify factors enabling or constraining progress. The remainder of the paper examines findings from the three countries and highlights policy implications and directions for future research.

### 1.1 UHC: conceptual framework and global context

UHC, introduced by the WHO in 2010, aims to ensure equitable access to essential health services—from promotion and prevention to treatment and rehabilitation—without financial hardship [8]. Conceptually, UHC comprises three dimensions: service coverage, population coverage, and the level of financial protection (FP). Countries typically progress by expanding covered services, extending eligibility to excluded groups, and reducing cost-sharing.

Global experience shows diverse pathways toward UHC. Some LMICs have prioritized FP through measures such as removing user fees or strengthening primary care (e.g., Zambia), while others have adopted rights-based approaches emphasizing legal entitlements and budget transfers to the uninsured (e.g., Latin America) [9]. Despite these reforms, nearly half of the world's population still lacks access to essential health services, and about two billion people face financial hardship from health spending [10], [11]. Persistent challenges—including allocation of financial resources, weak governance, and inefficient service delivery [12]—highlight the need to examine context-specific drivers and barriers to UHC in regions such as the MENA region.

### 1.2 Measuring and classifying UHC progress

This study uses the SCI, introduced by WHO in its global monitoring report [13] as part of SDG tracking, as the primary and routinely published measure, enabling cross-country comparability (see Section 2.1 for details). While the SCI is part of a broader composite UHC Index alongside FP index, it provides the most standardized basis for analysis. FP is commonly assessed through two indicators of out-of-pocket (OOP) spending: catastrophic expenditure (CATA) (OOP >10% of household consumption) and impoverishing expenditure (IMPOV) (OOP pushing households below the poverty line) [6].

The SCI measures coverage across four domains (SDG 3.8.1) [13]:

1. **Reproductive, maternal, newborn, and child health** (RMNCH) (e.g., antenatal care, family planning, DTP vaccination)
2. **Communicable diseases** (e.g., TB, HIV, malaria, sanitation)
3. **Non-communicable diseases** (NCDs) (e.g., hypertension, diabetes management)
4. **Service capacity and access** (e.g., workforce, hospital beds, emergency readiness)

These indicators enable benchmarking and classification of countries into low-, middle-, and high-performing UHC groups, which guided country selection for this study.

Table 1 below illustrates MENA's countries range of income levels, health expenditure, as well as SCI and FP indexes. Table A.1 in the Appendix provides further details on SCI scores.

**Table 1:**
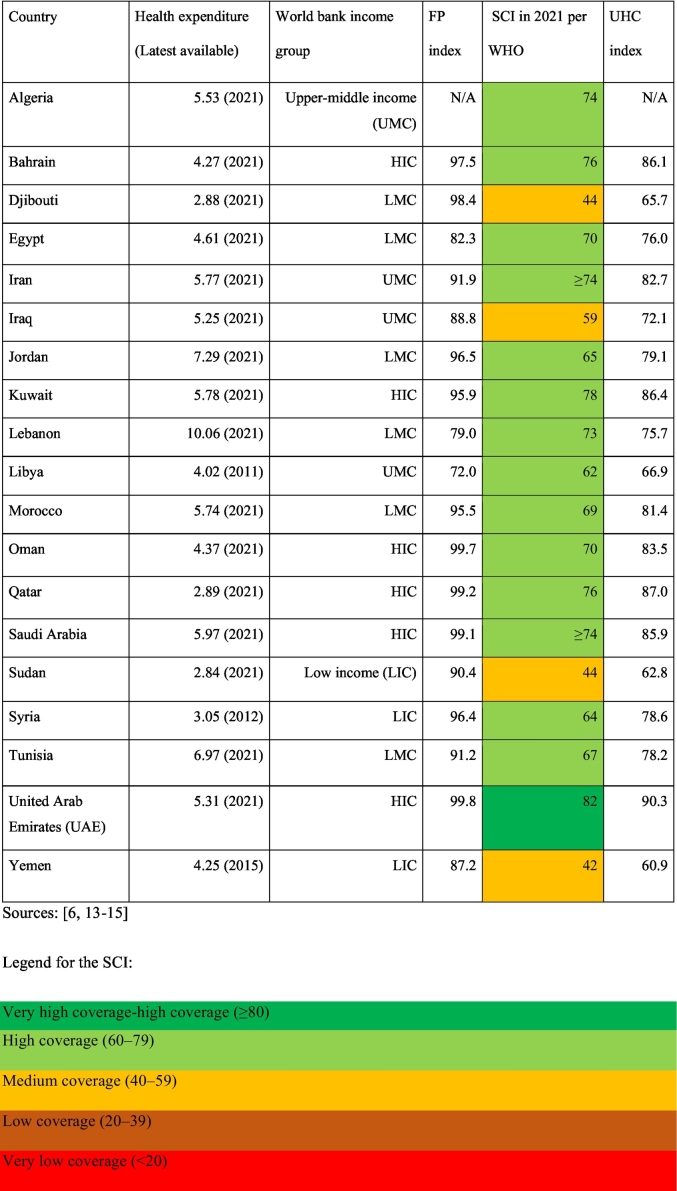
Countries' profiles and UHC scores.

Note: The legend was adapted for the MENA context. For instance, Yemen has the lowest score in the region (42), but it still falls within the ‘medium SCI’ category. The score is low relative to other MENA countries, not the legend itself.

### 1.3 Challenges to UHC in MENA

The literature documents the challenges in the progress toward UHC across the MENA region, related to financing, service delivery, governance, and political stability. It shows that public health spending is consistently below global averages, limiting fiscal space for expanding essential services [7]. Weak tax capacity and widespread informal employment are cited as barriers constraining domestic revenue mobilization and undermining equitable financing. As a result, OOP spending persists, increasing the risk of IMPOV [4], [5].

Studies show that service delivery weaknesses also impede UHC progress. Primary health care systems (PHC) are often underdeveloped and poorly integrated with higher levels of care, leading to inefficiencies and fragmented service pathways [3], [7]. Studies show that MENA health systems struggle with the quality of care as well as NCDs that affect their ability to achieve UHC [2].

Governance deficits and political instability are shown as structural barriers, with conflict, economic shocks, and institutional fragility disrupting reforms, diverting resources, and weakening regulatory capacity. The literature reports weak monitoring, limited accountability, and constrained civil society engagement as ongoing challenges to policy continuity and system oversight [4].

While the literature highlights these shared challenges, it often treats the region as homogeneous, overlooking substantial variation in governance, fiscal capacity, and institutional contexts. This study addresses this gap by examining one country from each of three UHC performance tiers—high, middle, and low—based on SCI scores. Applying a differentiated context-sensitive lens, it moves beyond a narrow focus on financing to suggest governance and sustained political commitment as key drivers of UHC progress in the MENA region.

## 2 Research design

### 2.1 Case selection

Based on the SCI scores (described above in Table 1), MENA countries were categorized into three performance tiers: very high to high performers, medium performers, and low performers, as shown in Table 2.

**Table 2:** Categorization Per SCI in 2021.

Very high performers to high performers		Medium performers	Low performers
UAE	Oman	Jordan	Yemen
Qatar	Iran	Egypt	Sudan
Kuwait	Algeria	Tunisia	Djibouti
Bahrain		Morocco	Libya
Saudi Arabia		Syria	
		Lebanon	

Three countries were purposively selected to represent these categories, based on: (1) diversity of income groups, (2) variation in SCI scores, and (3) inclusion of both extreme and mid-range performers. The UAE, as a HIC, represents the highest SCI score (82). Yemen, a LIC country, represents the lowest SCI score (42). Morocco, a LMIC with an SCI score of 69, represents a medium performer, closely aligning with the global average (68) and the regional average (66.4). Additionally, the authors' expertise in the Moroccan health system strengthens the scoping review's capacity to map challenges and opportunities in average-performing settings.

This selection shows how political, structural, and contextual factors influence UHC outcomes across performance tiers. By examining high-, medium-, and low-performing countries, the study captures both common challenges and context-specific drivers of UHC progress.

The SCI serves as the primary performance metric because it is the only routinely published and comparable UHC index reported by the WHO. Although FP (SDG 3.8.2) is a core UHC dimension, the WHO does not provide a single combined UHC index at the country level. SCI thus provides the most reliable and standardized basis for cross-country comparison. Literature constructing full UHC indices combining SCI and FP (e.g., Alshehari et al. [6]) is used to contextualize SCI performance and integrate FP dynamics, while SCI remains the guiding metric for case selection.

### 2.2 Methodology

A scoping review was conducted following the framework proposed by Arksey and O'Malley [16]. Among the four types of scoping review, this study adopted the third type, which synthesizes research findings for policymakers and practitioners who may lack resources for comprehensive reviews. This approach provides actionable insights on enablers and barriers to UHC in the MENA region, with a focus on the three selected case countries.

#### 2.2.1 Search strategy and data sources

The literature search drew on multiple electronic databases, including PubMed, Wiley, Cochrane Library, ScienceDirect, and Google Scholar. These databases cover peer-reviewed research on health. Google Scholar was included to capture grey literature and region-specific studies not indexed elsewhere. This approach ensured inclusion of documents reflecting national UHC experiences. Search terms combined keywords with Boolean operators:

(“Universal Health Coverage” OR UHC OR “health policy” OR “health reform” OR “healthcare system” OR “health system”) AND (barriers OR challenges OR progress), supplemented with country names. Searches initially targeted abstracts and were expanded to full texts as needed. A full overview of search strings is provided in Appendix-Table A.2.

#### 2.2.2 Inclusion criteria and screening

Eligible publications examined UHC progress, health system reforms, or system-level challenges in the UAE, Morocco, or Yemen, either through country-specific studies or regional analyses that included these countries, with a focus on key enablers and barriers. Only studies published from 2010—when WHO formally introduced the UHC framework—were included. Additionally, this timeframe reflects the aftermath of the Arab Spring, which heightened recognition of health as a human right [4]. The review examined studies on key reform priorities—chronic diseases, child mortality, and the health workforce—which reflect UHC challenges related to service delivery and health resources. Focusing on these areas highlights both structural and programmatic aspects of UHC implementation. Exclusions applied to studies published before 2010 or addressing UHC without a country-specific focus.

**Fig. 1:**
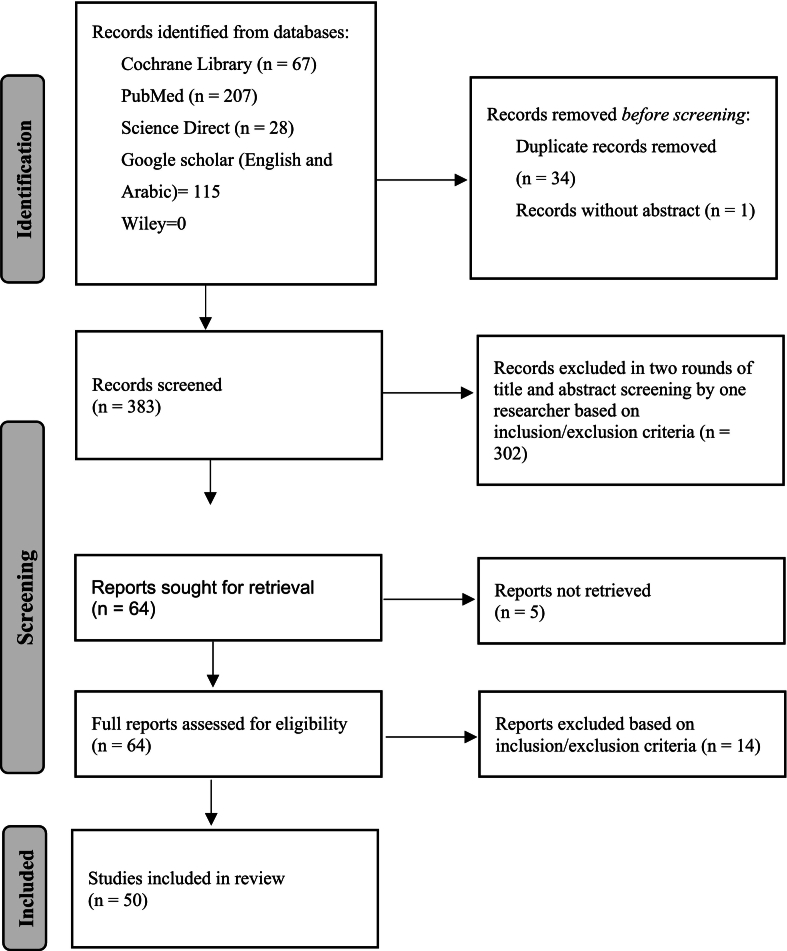
Identification of literature via databases. Source: [17].

The initial search retrieved 417 articles. After two-stage screening (title/abstract, then full text), 50 articles were retained: 16 articles were identified for UAE, 17 for Yemen, and 20 for Morocco. Fig. 1 summarizes this process. It is crucial to note that sum of documents for each country is higher than the total identified because some sources indicating the whole region were used for more than one country; specifically [5], [7] were used for both Morocco and Yemen.

#### 2.2.3 Data extraction

A standardized data extraction template (Appendix-Table A.3) was used to extract information, capturing metadata (reviewer, reference, year, study design, publication type) and the study's content, including country context and potential enablers and barriers to UHC.

#### 2.2.4 Analytical framework and data analysis

The review applied six dimensions derived from existing frameworks developed by Derakhshani et al. [18] and Darrudi et al. [19]. The former identified structural enablers and barriers to UHC through a systematic review, while the latter categorized challenges according to health system functions. This study groups them into two overarching categories (detailed in Table 3):

1. **Socio-economic conditions**
I. Social infrastructure and sustainability
II. Financial and economic infrastructure
2. **Health system–related dimensions**
I. Health stewardship and governance
II. Health resources
III. Service delivery
IV. Health system financing

**Table 3:** Framework of UHC Enablers and Barriers.

Dimension	Examples of enablers and barriers
Socio-economic conditions	
**Social infrastructure and social sustainability**	- Literacy level - Poverty level - Unemployment rate - Population's demographics
**Financial and Economic Infrastructures**	- GDP status - Growth level - GDP to health expenditure
Health system-related	
**Health-Stewardship/ governance dimension**	- Political will - Inter-sectoral coordination and supervisory capacity - Use of health information systems - Design of a benefit package, considering service and cost - Managerial inefficiencies in the insurance scheme - key stakeholders' support - Level of trust in public facilities
**Health Resources**	- Status of human resources - Health workers' distribution - Lack of/presence of technology, equipment and material
**Service delivery**	- Geographical access - Expansion of health coverage - Lack of recognizing priority demands - Quality of care - Status of basic care (i.e., immunization) - Increasing burdens of NCDs - Healthcare equity
**Health system financing- Revenue collection**	- Financial sustainability - Funding for PHC - Fragmentation of financing arrangements - Mismatches between the allocation of subsidies
**Health system financing- Pooling**	- Health insurance coverage for people and services - Fragmentation in health insurance funds - FP - Disproportionate co-payments
**Health system financing-Purchasing**	- Reimbursement for health expenses - Utilization of funds allocated to public health measures - Investment in health and PHC - Cost control

Sources: Author's own compilation guided by Derakhshani et al. [18] and Darrudi et al. [19].

This framework aligns with WHO's core requirements for UHC—efficient health systems, equitable financing, essential resources, and a competent workforce [8]. By distinguishing service delivery from resource creation, it allows clearer conceptualization of implementation barriers and enablers relevant in conflict contexts. Population coverage is not a standalone indicator, as it is embedded within service delivery, and governance, while population health is treated as an outcome of interactions of some or all these dimensions. Applying this framework provides a nuanced, context-sensitive understanding of how enablers and barriers shape UHC progress in the selected countries, with findings relevant to policymakers and health system planners.

Data analysis was conducted thematically, with extracted data categorized under the six dimensions to reflect MENA-specific political, economic, and health system contexts. To ensure quality, a second reviewer independently coded 10% of the data, with discrepancies resolved through discussion, guided by the analytical framework to identify recurring patterns and country-specific factors influencing UHC progress.

## 3 Results

Each country's subsection begins with contextual background, followed by analysis of enablers and barriers across the six dimensions.The number of studies for enablers and barriers across dimensions is presented in Appendix A (Tables A.4 and A.5)).

### 3.1 UAE

The UAE was established in 1971 as a federation of seven Emirates; Abu Dhabi, Dubai, Ajman, Umm Al Quwain, Sharjah, Fujairah and Ras Al Khaimah [20].

#### 3.1.1 Enablers

##### 3.1.1.1 Health stewardship

Eleven studies [20], [21], [22], [23], [24], [25], [26], [27], [28], [29], [30] identify political will prioritizing citizens' welfare and social development as a central enabler since state formation. Five studies [20], [21], [22], [23], [27] describe *UAE National Agenda 2021*, which articulated seven strategic pillars including strengthening the healthcare system and improving public health outcomes. Ahmad et al. [23] highlights the government's bottom-up approach, emphasizing the pivotal role of leadership in supporting and advancing policy agendas.

Two studies [20], [26] report complementary national plans introduced in 2017 targeting NCDs, mental health, nutrition, and maternal and child health. Together, these frameworks align national development strategies with population health priorities. Additionally, two studies [26], [30] associate cross-sectoral initiatives addressing waterborne diseases, delivery complications, and prenatal and postnatal care with reductions in child mortality.

##### 3.1.1.2 Economic infrastructure and social infrastructure

Seven studies [23], [24], [27], [28], [30], [31], [32] link economic growth—largely driven by oil revenues—to expanded health financing and infrastructure investment. Between 2000 and 2012, health expenditure as a share of GDP increased by over 36%, rising from US$2.3 billion to US$13.6 billion by 2014, with projections reaching US$25.7 billion in 2024 [28].

Few studies show relations between literacy and income with health outcomes. One study (Salam et al. [30]) reports that higher literacy, declining fertility, and women's empowerment improved maternal health awareness and maternal and child health outcomes. Another study (Malaviya et al. [33]) shows that the high income of nationals supports their increased utilization of health services.

##### 3.1.1.3 Service delivery

Service delivery witnessed enhancements in primary care. Al Ali and Al Sharif [26] note nationwide primary healthcare expansion with fewer than 200 residents living more than 30 km from a PHC center. Two studies [26], [29] link medicalized childbirth, expanded primary care, neonatal clinics, cancer screening, and breastfeeding initiatives to improved life expectancy and reduced mortality. Al Ali and Al Sharif [26] further report national breastfeeding and immunization programs reaching over 90% of children strengthened population health.

Six studies [21], [25], [26], [28], [29], [34] report sustained efforts to improve quality of care. Koornneef et al. [28] show that health facilities are internationally accredited. They report high patient satisfaction for services compared to other countries. Paulo et al. [20] report that Abu Dhabi reforms, including *the Patient-Centered Medical Home model* implemented in 2013, improved chronic disease management and reduced hospital admissions.

Health insurance expansion is documented in five studies [24], [27], [28], [33], [34]. Alshamsan et al. [34] describe a tiered insurance structure in which public schemes primarily cover citizens and private schemes serve expatriates. Malaviya et al. [33] report *Dubai's mandatory Insurance System of Advancing Health in Dubai (ISAHD) law* implemented in 2013 and effective in 2016. While Koornneef et al. [28] describe Abu Dhabi's three insurance plans including *Thiqa Plan* for citizens and two expatriate plans: *Basic* for low paid expatriates and *Enhanced* for high skilled expatriates. The literature provides no information on health insurance in other emirates, likely because Dubai and Abu Dhabi are the largest in terms of population and economic activity.

##### 3.1.1.4 Health financing

Four studies [21], [24], [28], [33] report FP measures supporting UHC. Koornneef et al. [28] show that overall OOP spending is about 20%, close to the OECD average. Malaviya et al. [33] show OOP expenditure in Dubai fell from 25% in 2014 to 13% in 2017 after *ISAHD* reform. Koornneef et al. [28] report over 22 million claims totaling US$2.9 billion in Abu Dhabi in 2014.

##### 3.1.1.5 Health resources

Three studies [26], [28], [35] showcase infrastructure expansion, including increased hospitals and clinics, with the increase of nurses and physicians by five-fold, particularly supporting primary care demand. Furthermore, three studies [21], [26], [35] highlight investment in integrated electronic medical records. Al-Ramlawi [21] reports telemedicine expansion improved patient–provider communication.

#### 3.1.2 Barriers

##### 3.1.2.1 Social infrastructure and service delivery

Despite these substantial achievements, six studies [20], [25], [26], [27], [31], [35] identify a rising burden of NCDs, including cardiovascular disease, obesity, and diabetes. Hajat et al. [31] highlight the government's Weqaya screening initiative, which established national NCD epidemiological profiles and supports prevention, early detection, and treatment strategies, particularly for cardiovascular conditions.

Six studies [20], [25], [29], [33], [34], [35] show that expatriates constitute most of the population, about 89% of residents. Four studies [25], [28], [33], [34] tackle the barriers facing low-skilled expatriates. Malaviya et al. [33] displays data from the 2014 and 2018 Dubai Household Health Surveys, showing higher services' utilization among white-collar nationals and residents compared with blue-collar expatriates.Similarly, Koornneef et al. [28] show that citizens access outpatient services approximately once per month, whereas expatriates use them three to four times less frequently. Two other studies [25], [34] indicate that low-skilled expatriates face high OOP. These disparities indicate a structural tension in the health system, where high population coverage coexists with unequal effective access. Reported barriers include language and cultural differences, administrative logistics, higher private insurance costs, and fear of deportation [33].

##### 3.1.2.2 Health resources

Workforce capacity also remains a concern. Shortages of specialized personnel are reported by two studies [32], [35]. Although expatriates support the health sector as providers, three studies [20], [28], [35] report high workforce turnover due to temporary residency, raising sustainability concerns for specialized care.

In summary, the UAE's progress toward UHC reflects sustained political commitment, economic investment, insurance expansion, and quality-focused service delivery. However, addressing rising NCDs' burden, citizen–expatriate inequities, and workforce sustainability remain key priorities for maintaining equitable coverage.

### 3.2 Morocco

Morocco, a constitutional monarchy in North Africa, had a population of 36.9 million in 2020, with 62% living in urban areas [36].

#### 3.2.1 Enablers

##### 3.2.1.1 Health stewardship

Eight studies [36], [37], [38], [39], [40], [41], [42], [43] identify strong political will as a central driver of stewardship. Six studies [7], [37], [39], [41], [42], [44] describe *the 2002 reform (Law No. 65–00),* which introduced *Compulsory Health Insurance (AMO)* for salaried workers, pensioners, students, and veterans, alongside *The Medical Assistance Scheme (RAMED)* for low-income populations. Two studies [5], [45] further report the INAYA scheme for the self-employed started in 2008. Three studies [36], [38], [40], [45] note that *The 2011 Constitution* reinforced commitment to healthcare and embedded UHC principles within seven of its articles.

Two studies [38], [39] report additional initiatives, including *The National Health Plan 2012–2016*, pharmaceutical reforms, and *The National Health Financing Strategy 2021* alongside people-centered programs targeting the elderly, persons with disabilities, prisoners, drug users, and cancer or HIV/AIDS patients. Belghiti Alaoui [38] also highlighted *The Advanced Regionalisation Law No. 111–14 (2015)* to reduce territorial inequalities, and the introduction of digital platforms *MAWIIDI* (appointments) and *CHIKAYA* (complaints) in 2016.

Bolan et al. [40] reported a dedicated US$8 million investment in health system strengthening beyond disease-specific programs. These reforms, led by the Ministry of Health (MOH) with WHO support, were anchored in *The Health Sectoral Strategy 2012–2016*, emphasizing PHC as the basis for UHC. *The 2013 National Health Conference* further promoted citizen and stakeholder engagement in the reform process.

One study (Elomrani et al. [36]) documented that health system strengthening improved population health outcomes: between 2000 and 2018, maternal mortality declined by 68% and neonatal mortality by 52%. This was supported by expanded antenatal care, institutional deliveries, and reduced socioeconomic disparities.

##### 3.2.1.2 Social and economic infrastructure

Elomrani et al. [36] reported sustained socioeconomic development, including improved income, electricity, sanitation, transport, and communication infrastructure. They show that women's education and empowerment further improved reproductive health awareness and outcomes. The same study highlight health expenditure's increasefrom US$61 to US$192 per capita between 1997 and 2018.

##### 3.2.1.3 Service delivery and health resources

Kabakian-Khasholian et al. [46] indicated that advancements in population’ health is attributed to the provision of RMNCH services at the primary care level. In addition to maternity care services, STIs/RTIs screening including HIV counselling and treatment, diagnosis and treatment, cervical cancer and breast cancer screenings, family planning services, are part of this package. While Elomrani et al. [36] show that the expansion of health facilities and increasing the number of midwives further improved maternal health.

#### 3.2.2 Barriers

##### 3.2.2.1 Health stewardship

Despite political will of the government, three studies [40], [45], [47] examine structural weaknesses in health governance. Tinasti [45] finds that governance is highly centralized, with decision-making concentrated in the MOH at the capital level and identifies corruption as a persistent issue. Bolan et al. [40] points to limited inclusion of private and community stakeholders in health planning processes. Meanwhile, Oufkir and Oufkir [47] show that although the government initiated a national health management information system, its implementation remains fragmented and delayed. This is largely due to poor coordination across health facilities, which has hindered the adoption of interoperability standards.

##### 3.2.2.2 Economic infrastructure

Cottin [48] notes that Morocco's GDP is below the MENA average. Tinasti [45] shows that in 2012 the government faced significant economic challenges: a fiscal deficit of 6%, and central government debt reaching 59.6% of GDP. Moreover, five studies [5], [42], [44], [45], [48] indicate relatively low public health spending. The government health expenditure per capita ($446.6 in 2014) remained below MENA ($712) and middle-income averages ($581) [48]. Total health spending stood at 6.5% of GDP, declining to 5.8% in 2013 [42].

##### 3.2.2.3 Service delivery

Eight studies [40], [42], [44], [49], [50], [51], [52], [53] report persistent inequities in healthcare access. Al Hassini et al. [49] describe rural–urban disparities linked to workforce concentration in cities, while Zahidi et al. [44] link these disparities to transportation barriers in remote areas. Cheikh et al. [52] identify inequities between public and private sector workers under AMO due to differences in reimbursement rules. *Health Insurance Fund for Civil Servants (CNOPS)* for public employees offers broader benefits than *Health Insurance Fund* for *Formal Sector Salaried Workers (CNSS)* for private employees, producing fragmented coverage and weak FP. Zekaoui et al. [50] report access barriers for women with disabilities due to infrastructure and transport limitations. Two studies [40], [51] showcase that prisoners, asylum seekers, and migrants face language and awareness barriers despite formal entitlement. Additionally, quality concerns were reported in nine studies [7], [36], [38], [40], [41], [42], [44], [45], [48], particularly in public services and among migrants [40], [45].

##### 3.2.2.4 Health financing

Fragmentation, due to multiple schemes and funds (CNOPS, CNSS, and RAMED) contributes to high OOP expenditure, reported in six studies [5], [7], [41], [44], [48], [52], reaching nearly 50% of total health expenditure (≈27.8 billion dirhams). During COVID-19, weaknesses in social protection prompted reform, and three studies [40], [43], [46] report the ongoing generalization of mandatory health insurance (2023) to unify different schemes.

##### 3.2.2.5 Health resources

Six studies [38], [40], [41], [42], [44], [45] report shortages of health personnel and low physician density (7.3 per 10,000).Specifically, three studies [44], [48], [49] show marked regional maldistribution, with higher physician density in Rabat-Salé-Kenitra, Fes-Meknes, and Casablanca-Settat, and the lowest in Beni Mellal-Khénifra and Drâa-Tafilalet.

No study was found to have examined causes of underinvestment in workforce training and infrastructure. Most remained descriptive, reporting shortages, while discussing organizational (e.g., low investment in training and education) or personal (e.g., burnout/stress) challenges, without analysing governance or financing determinants [49]. The only recurring explanation (Mataria et al. [7]) relates to reimbursement incentives in the mixed public–private system favouring service provision over long-term investment. Overall, evidence remains insufficient to determine whether gaps stem from fiscal constraints, governance inefficiencies, or policy priorities.

In short, Morocco demonstrates strong political commitment to expanding coverage and improving population's health. Nevertheless, structural weaknesses in governance combined with service delivery and resource constraints create a persistent gap between legal entitlement and actual access, especially for vulnerable groups. Enhancing governance centralization, reducing geographic and socioeconomic inequities, and prioritizing investment in the health workforce remain key priorities for advancing UHC.

### 3.3 Yemen

Yemen, one of the poorest countries in the MENA region, faced major economic and social challenges even before the 2011 Arab Spring [54]. Conditions deteriorated further in 2014 when civil war erupted after the Houthi militia seized the capital, triggering a Saudi-led coalition intervention [55]. The conflict severely disrupted public services, including the health sector, and intensified pre-existing system weaknesses, limiting progress toward UHC [54].

#### 3.3.1 Potential enabler

##### 3.3.1.1 Service delivery

Some attempts were made to improve RMCNH services. Two studies [54], [55] report initiatives introduced during the conflict. The United Nations Population Fund (UNFPA) developed *The Minimum Initial Service Package* (MISP), aimed to standardize the provision of RMCNH services in emergency settings [55]. In Hodeida governorate, the MOH collaborated with the WHO to improve access to services, particularly reproductive care [54]. However, these efforts were constrained by the numerous systemic barriers outlined below.

#### 3.3.2 Barriers

##### 3.3.2.1 Health stewardship

Governance challenges, undermining UHC progress, are reported across nine studies [4], [54], [55], [56], [57], [58], [59], [60], [61]. Specifically, weak governance is indicated in three studies [54], [55], [56], while widespread corruption and low public trust are reported in five studies [4], [54], [55], [58], [61]. Six studies [54], [55], [59], [60], [61], [62] document poor coordination and administrative fragmentation. Fragmentation occurs across political, institutional, financial, and strategic dimensions due to the existence of two governments with separate ministries [59], [60]. Competing health ministries weaken policy implementation [7], [56], and intersectoral fragmentation arises as the Defense and Interior ministries provide parallel healthcare services [59]. Additionally, dependence on donor funding, reported in three studies [54], [57], [59], further misaligns national priorities, separating health security activities from broader UHC goals. Ongoing conflict continues to damage already fragile infrastructure [54], [57], [59].

##### 3.3.2.2 Social and economic infrastructure

Socio-economic conditions remain extremely constrained. Very high poverty levels—approaching 80% are reported in four studies [54], [55], [59], [60]. Low literacy rates of about 54.1% are documented in eight studies [3], [54], [55], [56], [59], [60], [61], [63]. Dureab et al. [60] indicated that high poverty rates combined with political instability led to the deterioration of population health outcomes. Four studies [56], [58], [59] report that approximately 2.2 million children are acutely malnourished, including 462,000 with severe acute malnutrition, substantially increasing child mortality.

Additionally, health expenditure represents only 4% of GDP, while low government contribution to health financing, declining from 54% to 23% in 2014, is reported in four studies [3], [4], [54], [64]. These contextual constraints hinder basic system functioning.

##### 3.3.2.3 Health financing

Pooling and FP remain weak. Holst and Gericke [64] report that before 2011, the country operated an inefficient four-tiered system alongside an unregulated private sector. Nine studies [3], [7], [54], [55], [56], [57], [58], [64], [65] describe rising OOP spending. Before the conflict, 17% of households experienced CATA and 2.4% spent more than 25% of their income on healthcare [54]. User fees introduced in the 1990s escalated informally, leading to extremely high OOP spending—71% in 2011 [4] and over 80% by 2015 [65].

##### 3.3.2.4 Service delivery

Service delivery constraints existed even before the conflict. Two studies [54], [55] reveal that Yemen lacked a clearly defined essential service package—guided by external funds rather than actual population needs—and never implemented a national insurance system. This left 65% of the population uninsured [55]. Furthermore, Garran [55] indicates that geographic isolation is a major barrier to healthcare access, with long distances and the fact that roughly 80% of roads are unpaved significantly hindering access to health services. This resulted in high maternal mortality rates, particularly among adolescents, women of low socioeconomic status, and residents of conflict-affected regions, as reported by two studies [56], [58].

Healthcare quality is low, particularly in curative and maternity care, as reported in seven studies [4], [54], [55], [56], [57], [58], [64]. Five studies [4], [58], [59], [60], [66] indicate minimal investment in primary care. The latter studies show that coverage gaps are evident in immunization services: only 35% of functioning facilities provide vaccination, contributing to outbreaks such as diphtheria, where 46% of cases and 69% of deaths occurred among unvaccinated children [60].

##### 3.3.2.5 Health resources

Ten studies [55], [56], [57], [58], [59], [60], [61], [63], [64], [65] report limited infrastructure capacity: only 54% of facilities are fully operational, 40% partially functional, and 6% non-functional due to damage and fuel and electricity shortages. Resource shortages, especially medicines, are also widely reported in ten studies [4], [5], [6], [7], [8], [9], [10], [11], [12], [13], [14], [15], [16], [17], [18], [19], [20], [21], [22], [23], [24], [25], [26], [27], [28], [29], [30], [31], [32], [33], [34], [35], [36], [37], [38], [39], [40], [41], [42], [43], [44], [45], [46], [47], [48], [49], [50], [51], [52], [53], [54], [55], [56], [58], [59], [60], [61], [66]. Two studies [54], [56] link medicine shortages to storage limitations, port closures, and economic collapse further disrupt care.

Workforce shortages are severe as reported in five studies [55], [57], [58], [59], [61]. These studies further show uneven rural distribution of health workers, creating major access inequities. Three studies [55], [56], [58] specifically report shortages of female physicians, limiting women's access to care.

In short, Yemen illustrates how conflict magnifies pre-existing system weaknesses, creating barriers across all UHC dimensions. The country faces pervasive poverty, weak governance, fragmented service delivery, insufficient resources, and minimal FP. In this context, restoring security and basic service functionality is a prerequisite for meaningful progress toward UHC.

Table 4 below summarizes the main enablers and barriers found across the three countries in accordance with the six dimensions.

**Table 4:** Summary of Health System Enablers and Barriers by Dimension (UAE, Morocco, Yemen).

Dimension	UAE	Morocco	Yemen
Social infrastructure	E: High literacy linked to better outcomes B: Demographic(majority of expatriates)	E: improved infrastructure and income, women's empowerment B: —	E: — B: Low literacy, high poverty
Economic infrastructure	E: Economic growth and increased health expenditure B: —	E: Socioeconomic development B: Low health expenditure	E: — B: Very low health expenditure
Health stewardship	E: Strong political will; national health agenda/initiatives B: —	E: National initiatives, Health reforms (AMO, RAMED). B: centralization, fragmentation	E: — B: Weak/fragmented governance post-conflict; absence of health insurance pre-conflict
Service delivery	E: High-quality primary care; expanded coverage to citizens & expatriates B: Inequities between citizens and expatriates	E: Coverage expanded via AMO/RAMED. B: Geographic/sectoral inequities, low quality of care	E: Initiatives with UNFPA and WHO B: lack of investment in PHC especially vaccination, low quality of care
Health resources	E: — B: Limited specialized care; unsustainable expatriate workforce	E: — B: Staff shortages; maldistribution (urban–rural)	E: — B: workforce and severe medicines shortages; damaged infrastructure (∼45% functional) due to conflict
Health financing	E: Low OOP B: Fragmented insurance; inter-emirate differences (lower in Dubai than Abu Dhabi).	E. generalization of health insurance (recent) B: High OOP	E: — B: Very high OOP

Abbreviations: E = Enablers; B = Barriers, – = None.

Source: Authors' own compilation.

## 4 Discussion

### 4.1 Synthesis of findings

This study suggests that progress toward UHC in the MENA region reflects more than financial capacity alone. The reviewed literature indicates that governance structures, political stability, socio-economic conditions, and health system functions interact in shaping UHC trajectories across the region. Although national income is positively associated with UHC performance in the MENA region [3], [6], [7], global evidence [9], [10], [11], [12] indicates that economic wealth alone is insufficient to achieve effective health coverage. Governance structures critically shape how financial and institutional resources are translated into accessible, equitable services. Systems with well-defined rules, robust accountability mechanisms, and inclusive implementation tend to realize faster improvements in coverage, equity, and quality of care. Evidence from the UAE, Morocco, and Yemen further underscores that income alone does not determine UHC trajectories; rather, effective stewardship, institutional coordination, and the translation of political commitments into sustained reforms emerge as pivotal, positioning governance mechanisms themselves as central determinants of UHC.

The six-dimension analytical framework applied in this study helps illuminate several cross-cutting themes. First, across the three contexts, governance functions as both an enabler and a constraint. In the UAE, early political commitment and leadership support, combined with a bottom-up planning approach [20], [21], [22], [23], [24], [25], [26], [27], [28], [29], [30], have fostered a well-resourced and coordinated health system. This aligns with literature showing that stewardship facilitates effective purchasing, quality assurance mechanisms, and strategic service expansion [21], [23], [28].

In Morocco, political will has supported the expansion of health coverage through RAMED and AMO [7], [37], [39], [41], [42], [44]; however, several studies [33], [36], [39], [40], [41], [49] indicate that this has not automatically translated into equitable access. Structural inequities—geographic disparities, differing health needs, and income-based barriers—combined with fragmentation between schemes and uneven service quality, mean that some populations benefit less than others. Additionally, governance challenges—centralization, corruption, and limited communication across health facilities [40], [45], [47]—highlight that effective stewardship and coordination are crucial determinants of whether policy intentions result in actual, equitable UHC outcomes. As a result, a gap between policy intentions and implementation has occurred despite formal coverage gains. Similar patterns are observed in LMICs globally, where coverage expansion is not matched by health workforce development or strong FP, resulting in persistent OOP expenditures and inconsistent entitlements [5], [11].

Yemen's experience further illustrates how political instability and institutional fragmentation (two different governments with different health ministries) weaken health system performance across all dimensions. The collapse of infrastructure, loss of pooling mechanisms, and reliance on external actors mirror patterns observed in fragile and conflict-affected countries, where instability undermines governance and erodes progress toward UHC [55], [59], [62].

Second, social infrastructure—particularly education, poverty, and demographic pressures—shapes both service demand and the effectiveness of preventive care measures. In the UAE, high literacy levels and demographic transition support uptake of screening and maternal health services [30] and high income supported utilization of services [33]. In Morocco, reductions in maternal and neonatal mortality between 2000 and 2018—by 65% and 52%, respectively—have been associated with improvements in women's education and access to reproductive services [36]. In Yemen, specific literature [60] links high levels of poverty with poor health outcomes, reinforcing global evidence that social determinants are critical for advancing UHC in low-income settings [10].

Third, economic infrastructure, reflected in sustained economic growth, has enabled higher healthcare spending in the UAE [23], [24], [27], [28], [30], [31], [32], while Yemen and Morocco continue to face low health expenditure [3], [4], [5], [42], [44], [45], [48], [54], [64]. This disparity constrains investment in infrastructure and contributes to persistent workforce shortages [49]. However, the literature does not report on the reasons for lack of investment in healthcare.

Fourth, health resource enablers include expanded infrastructure in the UAE and Morocco, supporting increased primary care demand [26], [28], [35], [36], as well as the digitalization of healthcare in the UAE [21], [26], [35]. Nonetheless, health workforce shortages and maldistribution persist across the region. Morocco and Yemen face pronounced deficits in rural and remote areas, as shown in [44], [48], [55], [57], [58], [59], [61], while the UAE contends with heavy reliance on expatriate workers, raising concerns about long-term sustainability [20], [28], [34]. This supports regional analyses that show workforce constraints as a key limitation on service expansion and quality [3], [5].

Fifth, FP remains a persistent challenge, particularly in the cases of Morocco and Yemen. The UAE has successfully reduced OOP spending to approximately 20%, comparable to OECD levels, although variations exist between Dubai and Abu Dhabi. In Abu Dhabi, low-skilled workers remain at risk of higher OOP due to limitations of the *Basic* insurance package [28], [33]. Morocco continues to rely heavily on household financing [5], [7], [41], [44], [48], [52]. Nonetheless, addressing insurance fragmentation through the generalization of health insurance [40], [43], [46] might reduce OOP in the future. Yemen's OOP share—exceeding 80%—is among the highest globally [3], [54], [55], [56], [57], [58], [64], [65]. These patterns reflect broader evidence that fragmented pooling arrangements and insufficient pre-payment mechanisms hinder equitable financing in many low and middle-income settings [8], [12].

Finally, enablers for services delivery indicate the importance of PHC strengthening. The UAE's robust PHC network—where fewer than 200 residents live more than 30 km from a PHC facility—alongside digital health innovations illustrate how strong PHC systems underpin UHC gains [22], [26]. In Morocco, efforts to increase antenatal care coverage and advancements in population’ health is attributed to the provision of RMNCH services at the primary care level [36], [46]. In Yemen, despite attempts of enhancing RMNCH services [54], [55], limited investment in PHC and in a basic benefits package prior to and during conflict has reduced system resilience, contributing to outbreaks of preventable diseases and persistent service disruptions [4], [54], [55], [57].

Nonetheless, barriers in service delivery show persistent inequities across all cases. In the UAE, inequities occur between citizens and expatriates, driven by limited coverage under basic insurance packages for low-skilled workers, as well as differences in age and income [25], [28], [33]. In Morocco, gaps exist across geographic areas, job sector, and vulnerable populations, compounded by unevenly allocated infrastructure that do not meet special needs [40], [42], [44], [49], [50], [51], [52], [53]. In Yemen, geographical barriers (long unpaved roads) are the main obstacle in accessing care [55].

### 4.2 Strengths of the study

A key strength of this study is its use of a structured analytical framework to synthesize diverse evidence across multiple health system dimensions. Drawing on literature from three countries at different stages of UHC progress, the review captures both shared regional patterns and context-specific challenges. This approach avoids presenting UHC as a uniform pathway and instead supports a more nuanced understanding of priorities aligned with country capacity.

Building on the conceptual insights of Derakhshani et al. [18] and the operational dimensions articulated by Darrudi et al. [19], the study reorganizes key UHC determinants into socio-economic and health system–related domains. This structure enables a clearer examination of how governance and stewardship mediate the relationships between financing, service delivery, and health resources across different MENA contexts. When applied to the regional literature, this framing highlights recurring dynamics shaping UHC trajectories, including political stability and governance fragmentation (Yemen), political will and enforcement of inclusive reforms (UAE), and reform and equity dimensions of reform implementation (Morocco).

Importantly, the framework offers analytical value beyond existing WHO models in three ways:

• It explicitly integrates contextual factors—such as governance, political stability, and socio-economic conditions—into the analysis of health system performance, rather than treating them as external or background influences.
• It contextualizes UHC pathways within the specific political and economic realities of the MENA region, highlighting cross-country variation that WHO models may not fully capture.
• It demonstrates how governance functions as a cross-cutting determinant of UHC by showing how fragmented governance structures shape system-level outcomes, shifting the explanation of UHC performance beyond financial capacity alone.

### 4.3 Policy implications

The findings point to several concrete policy implications across the framework dimensions. Governance is the foundational entry point for all other policy dimensions. Effective governance should extend beyond formal policy adoption to ensure enforcement, sustained political commitment, and accountability across health system functions. Strengthening social infrastructure requires targeted investment in education and health literacy to increase awareness of entitlements and improve service utilization, particularly among marginalized groups. In LMICs and LICs, economic infrastructure depends on allocating sufficient and predictable public funding to health despite competing sectoral demands and aligning spending with clear UHC priorities.

Service delivery reforms should focus on strengthening primary care, expanding and equitably distributing the health workforce, and investing in rural and remote infrastructure. Improving quality of care in the public sector is essential to build trust and reduce inequities. In countries where expatriates constitute the majority, services should be accessible near labor accommodations and industrial areas, alongside multilingual outreach to raise awareness of health rights—especially among low-skilled migrant workers facing legal or informational barriers. Digital tools, where feasible, can improve access and system efficiency. Health financing reforms should reduce fragmentation across multiple insurance pools and strengthen risk pooling to lower OOP spending and protect vulnerable groups. Optimizing health resources requires long-term workforce planning, retention incentives for rural areas, and stronger health information systems.

The prioritization of these policy actions should reflect country context and UHC progress. For example, HICs countries may focus on sustainability and NCD prevention. LMICs may emphasize reducing equity gaps and addressing system fragmentation. Fragile or conflict-affected countries should first restore basic service delivery and foundational health system functions before scaling broader reforms.

### 4.4 Limitations

Several limitations should be acknowledged when interpreting these findings. As a scoping review, the analysis is constrained by the availability and quality of published and grey literature, which may not fully capture unpublished policy documents or implementation processes. Specifically, the available literature on Yemen is heavily skewed toward barriers and crisis conditions, which may underrepresent locally driven coping mechanisms or incremental service adaptations that are less frequently documented in published sources. The inclusion of three countries is intended as an illustrative approach rather than a formal comparative case study, and findings are therefore may not be generalizable to the entire MENA region. Variability in reporting quality—especially where health information systems are weak or fragmented—may also have influenced interpretation.

Additionally, while the six-dimension analytical framework provided structure, categorizing complex health system factors inevitably involves some degree of subjectivity, particularly in distinguishing between ‘governance’ and ‘stewardship’ or common issues between ‘service delivery’ and ‘health resources’. Although FP indicators were reported and analyzed within the country cases, the initial classification of countries into UHC performance tiers relied primarily on SCI scores. This may have influenced case selection and grouping which may not fully reflect cross-country variation in equity dimensions of UHC at the classification stage.

Furthermore, the review relied primarily on English-language sources; although targeted searches were conducted in Arabic, no relevant UHC-focused publications were identified, and relevant non-English sources—particularly French-language literature for Morocco—may therefore have been missed. Nevertheless, the review offers valuable insights into recurring socio-economic and system-level enablers and barriers and highlights priority areas for future empirical research.

Overall, the reviewed evidence underscores that advancing UHC requires integrated, multisectoral strategies tailored to context. In contrast to regions such as Latin America and parts of Southeast Asia—where UHC expansion has often been supported by earlier social insurance development, strong PHC, and gradual fiscal integration—progress in the MENA region is shaped more strongly by political economy factors. These include state capacity, conflict exposure, and governance continuity. These dynamics make governance and stewardship particularly decisive in shaping UHC trajectories, at times outweighing financial capacity alone.

## 5 Conclusion

This study provides a structured framework for understanding how contextual and health system factors interact to shape UHC progress in the MENA region. This framework is most relevant for policymakers and reform designers responsible for health system planning and UHC-related reforms—particularly within MOH and parliamentary health committees in MENA countries. It may also be useful for development partners and donors operating in fragile and conflict-affected settings, as it highlights the importance of aligning external support with nationally identified system priorities rather than donor-driven agendas. It is also relevant for contexts where political economy factors—such as governance capacity, institutional fragmentation, and political stability—strongly shape UHC trajectories.

This scoping review synthesized evidence on enablers and barriers to UHC in the MENA region using a structured six-dimension analytical framework. Rather than a formal comparative analysis, it drew on literature from three contexts—the UAE, Morocco, and Yemen—to illustrate how UHC dynamics, shaped by socio-economic conditions and health system functions, vary across SCI performance levels.

The findings show that financial capacity alone does not drive UHC progress. Governance arrangements, political stability, and institutional coordination determine how resources translate into coverage, service quality, and FP. High-income settings highlight the role of strong stewardship, middle-income contexts show the need for accountableand communicative governance, and fragile settings demonstrate how instability and institutional fragmentation constrain even basic service delivery.

Cross-cutting themes emerging from the review include the importance of governance and stewardship, investments in PHC and workforce development, and attention to FP mechanisms. Social determinants—such as poverty, education, and demographic pressures—remain critical in shaping UHC outcomes. These findings point to the importance of adaptive governance systems that can respond to shifting needs and external shocks.

Future research should examine how reforms of governance and social contracts shape health system performance, with particular attention to mechanisms that promote equity, accountability, and sustainability. Such work would support the SDGs' call for integrated, cross-sectoral strategies and provide further insight into how coordinated policy approaches may strengthen progress toward UHC and broader societal wellbeing.

## Declaration of generative AI and AI-assisted technologies in the manuscript preparation process

During the prepartion of this work, the authors used ChatGPT for language editing and enhancing sentence structure. After using this tool, the authors reviewed and edited the content as needed and take full responsibility for the content of the published article.

## CRediT authorship contribution statement

**Mai Mohamed Abdou Mahmoud:** Writing – original draft, Methodology, Investigation, Formal analysis, Conceptualization. **Lorraine Frisina Doetter:** Writing – review & editing, Supervision, Investigation, Conceptualization.

## Funding

This work was supported by the 10.13039/501100001659Deutsche Forschungsgemeinschaft as part of the 10.13039/501100007837A04 Project of the Collaborative Research Centre 1342 at the University of Bremen, Germany.

## Declaration of competing interest

The authors declare no competing interests.
