# Supplementary material for: Enablers and barriers to MENA'S path to universal health coverage: A scoping review of UAE, Morocco and Yemen
Source: Dialogues Health. 2026 Mar 10;8:100291. doi: 10.1016/j.dialog.2026.100291 (PMC13049542; doi:10.1016/j.dialog.2026.100291)
Supplement: Supplementary file 1 — Supplementary material to this article can be found online at. [file mmc1.docx]

**Appendix A.**

**Table A.1.** Aggregate SCI for MENA Countries Per the Four Areas

| Country | RMNCH | Infectious diseases | NCDs | Service capacity and  access | UHC SCI (SDG 3.8.1) |
| --- | --- | --- | --- | --- | --- |
| Algeria | 68 | ≥80 | 61 | ≥80 | 74 |
| Bahrain | ≥80 | ≥80 | 57 | ≥80 | 76 |
| Djibouti | 52 | 35 | 58 | 35 | 44 |
| Egypt | ≥80 | 62 | 62 | 78 | 70 |
| Iran | ≥80 | 55 | 69 | 80 | ≥74 |
| Iraq | 61 | 54 | 64 | 55 | 59 |
| Jordan | 70 | 62 | 58 | 70 | 65 |
| Kuwait | ≥80 | ≥80 | ≥80 | 60 | 78 |
| Lebanon | 71 | ≥80 | 54 | ≥80 | 73 |
| Libya | 66 | 57 | 52 | 76 | 62 |
| Morocco | 75 | ≥80 | 57 | 65 | 69 |
| Oman | 63 | ≥80 | 58 | 78 | 70 |
| Qatar | ≥80 | ≥80 | 62 | ≥80 | 76 |
| Saudi Arabia | 75 | ≥80 | 49 | 80 | ≥74 |
| Sudan | 51 | 43 | 54 | 30 | 44 |
| Syria | 62 | 68 | 65 | 62 | 64 |
| Tunisia | ≥80 | 55 | 57 | 75 | 67 |
| UAE | ≥80 | ≥80 | 61 | ≥80 | ≥80 |
| Yemen | 41 | 46 | 62 | 28 | 42 |

Legend:

Very high coverage-high coverage (≥80)

High coverage (60–79)

Medium coverage (40–59)

Low coverage (20–39)

Very low coverage (<20)

Sources [13,14]

**Table A.2.** Search Strategy

| Databases | Search terms |
| --- | --- |
| PUBMED | ("Universal Health Coverage" OR UHC OR "health policy" OR “health reform" OR "healthcare system" OR "health system") AND (barriers OR challenges OR progress) AND ("United Arab Emirates"[Title/Abstract] OR UAE[Title/Abstract] OR Morocco [Title/Abstract] OR Yemen [Title/Abstract]) |
| Cochrane library | ("Universal Health Coverage" OR UHC OR "health policy" OR "health reform" OR "healthcare system" OR "health system") AND (barriers OR challenges OR progress) AND ("United Arab Emirates" OR UAE OR Morocco OR Yemen) in all text |
| Wiley | "("Universal Health Coverage" OR UHC OR "health policy" OR "health reform" OR "healthcare system" OR "health system") AND (barriers OR challenges OR progress) AND ("United Arab Emirates" OR UAE OR Morocco OR Yemen)" in Abstract |
| Google scholar in English | ("Universal Health Coverage" OR UHC OR "health policy" OR "health reform" OR "healthcare system" OR "health system") AND (barriers OR challenges OR progress) AND ("United Arab Emirates" OR UAE OR Morocco OR Yemen) for first 10 pages |
| Google Scholar in Arabic | ("التغطية الصحية الشاملة" OR "UHC" OR "السياسة الصحية" OR "إصلاح النظام الصحي" OR "نظام الرعاية الصحية" OR "النظام الصحي")  AND (العوائق OR التحديات OR التقدم)  AND ("الإمارات العربية المتحدة" OR "الإمارات" OR "المغرب" OR "اليمن") |
| Science Direct | ("Universal Health Coverage" OR UHC) AND (barriers OR challenges OR progress) AND ("United Arab Emirates" OR UAE OR Morocco OR Yemen) |
| Web of science | ("Universal Health Coverage" OR UHC) AND (barriers OR challenges OR progress) AND ("United Arab Emirates" OR UAE OR Morocco OR Yemen) |

**Table A.3.** Data Extraction Template for Enablers and Barriers

| Metadata | |
| --- | --- |
| Reviewer’s Initials |  |
| Article Reference (APA reference style) |  |
| Year of Publication |  |
| Article Type:  E.g., peer reviewed; grey literature |  |
| Abstract |  |
| Method used (Systematic Review, Scoping Review etc.) |  |
| Aim of the study |  |
| Which aspect of health policy is addressed? (e.g., NCDs, child mortality, tackling public health risks. etc) |  |
| Country Background | |
| Country/ies/region in study |  |
| General challenges faced (economic, societal or political)/context of the country |  |
| UHC Progress | |
| Enablers for UHC achievement  This can include  Economically:   - Economic growth   Health wise:   - Country’s attempts to reform its health system to increase coverage - Political will: priotrization of health agenda - Increasing health expenditure - Focus on primary care - Increasing equity (e.g., focus on vulnerable groups and increased FP |  |
| Barriers to UHC’s achievement  Health barriers   - Lack of investment in health - Lack of adequate health infrastructure, health personnel and - Poor health system functionality (either in governance, service delivery or financing)   Economic barriers:   - Poor economic situation (e.g., economic stagnation, inflation, growing informal sector   Political: conflicts or instabilities |  |
| Theme/main category of the  UHC barrier or enabler   - Social infrastructure and social sustainability dimensions - Financial and Economic Infrastructures dimensions - Service delivery - Health-Stewardship/ governance dimension - Health resources - Health financing |  |
| The study’s recommendations (if available) |  |
| Notes |  |

Source: Authors’ creation

**Table A.4**. Number of Studies on Enablers Across Dimensions

| Country | Health stewardship | Economic infrastructure | Social infrastructure | Service delivery | | | FP-OOP | Health resources |
| --- | --- | --- | --- | --- | --- | --- | --- | --- |
|  |  |  |  | Primary care | Expansion of health insurance | Quality of care |  |  |
| UAE | 11 | 7 | 2 | 2 | 5 | 6 | 4 | 3 |
| Morocco | 8 | 1 | 1 | 1 | 3 (generalization of health insurance) | _ | _ | 1 |
| Yemen | ­_ | _ | _ | 2 | _ | _ | _ | _ |

**Table A.5.** Number of Studies on Barriers Across Dimensions

| Country | Health stewardship | Economic infrastructure | Social infrastructure | Service delivery | | | FP-OOP | Health resources | |
| --- | --- | --- | --- | --- | --- | --- | --- | --- | --- |
|  |  |  |  | inequities | Quality  of care | Burden of NCDs |  |  |  |
|  |  |  |  |  |  |  |  | resources | Health workers |
| UAE | _ | _ | 6 (demographics) | 4 | _ | 6 | 1 | _ | 2 (specialized workforce)  3 (turnover) |
| Morocco | 3 | 5 (health spending)  2 (economic hurdles) | _ | 8 | 9 | _ | 6 | _ | 6 |
| Yemen | ­9 | 4 | 8 (literacy)  4 (poverty) | 3 | 7 |  | 9 | 10 | 10 |
